# 

**Published:** 1889-08

**Authors:** 


### CONTENTS—AUGUST, 1889.—VOL. 5, NO. 2.

ORIGINAL COMMUNICATIONS— Page.

Vis Medicatrix Naturæ. By J. W. Harrison, M. D . . . 43

Ligation of Subclavian and of Common Carotid Artery for Aneurisms. By W. J. Pettus, M. D . . . . . . . . 50

A Remarkable Case of Elephantiasis, By T. J. Bennett, M. D . . . . . . . . . . . . . . . . . . . . . 52

A Tarantula Bite. By J. C. Baird, M. D. . . . . . . . . 53

Minor Surgery. By D. L. Peeples, M. D. . . . . . . . . 57

CORRESPONDENCE—

Young Parents. E. McD. Bridgeford, M. D . . . . . . . 59

Improved Method of Artificial Preparation. By J. E. Thomas, M. D. . . . . . . . . . . . . . . . . . . 59

SOCIETY NOTES—

Central Texas Medical Society--Proceedings of the last meeting . . . . . . . . . . . . . . . . . . . . . 60

EDITORIAL—

Bush-Whacking . . . . . . . . . . . . . . . . . . . 63

The Latest Elixir |Vitæ . . . . . . . . . . . . . . . 65

The Rise and Fall of the P. M. B. A. . . . . . . . . . . 68

MEDICAL NEWS AND MISCELLANY—

Numerous Interesting Items . . . . . . . . . . . . . . 70

BOOK NOTICES AND REVIEWS—

Keating's "Cyclopedia of Dissases of Children" . . . . 72

PUBLISHERS' NOTES—

Of Interest to Our Readers . . . . . . . . . . . . . . 76

